# Diagnostic Challenge of Primary Hepatic PEComa in a Young Woman with Prior Colorectal Cancer

**DOI:** 10.14309/crj.0000000000002377

**Published:** 2026-11-04

**Authors:** Gerald Odong, Priyanka Panwar, Lavender Otom, Ivy Wakahora, Steven Idet, Sarah Kibe, Patricia Okiro, Saleem Abdulkarim, Ajua Alemanji, Amina Kidee, Samuel Gitau

**Affiliations:** 1 Department of Radiology, The Aga Khan University Hospital Nairobi, Nairobi, Kenya; 2 Department of Medicine, The Aga Khan University Hospital Nairobi, Nairobi, Kenya; 3 Department of Pathology, The Aga Khan University Hospital Nairobi, Nairobi, Kenya; 4 Department of Haemato-Oncology, The Aga Khan University Hospital Nairobi, Nairobi, Kenya

**Keywords:** Primary hepatic PEComa, hypervascular liver lesion, hepatocellular carcinoma mimic, immunohistochemistry, HMB45, mTOR inhibitors, perivascular epithelioid cell tumors

## Abstract

Primary hepatic perivascular epithelioid cell tumor (PEComa) is a rare mesenchymal neoplasm that can mimic common hepatic malignancies. A 28-year-old African woman with colorectal cancer in remission presented with 3 months of epigastric pain and tender hepatomegaly. Tumor markers were normal. Ultrasound and triphasic CT showed 2 large heterogeneous hypervascular hepatic masses with progressive enhancement and early venous drainage; hepatobiliary iminodiacetic acid scan (HIDA) SPECT-CT demonstrated photopenic lesions. Biopsy revealed epithelioid cells with focal HMB45, diffuse smooth muscle actin, and Ki-67 20%–30%, confirming hepatic PEComa. Resection was feasible but deferred; everolimus was started before loss to follow-up.

## INTRODUCTION

Perivascular epithelioid cell tumors (PEComas) are rare mesenchymal neoplasms composed of distinctive perivascular epithelioid cells with dual melanocytic and smooth muscle markers, first described by Bonetti and later recognized by the World Health Organization as a family of tumors characterized by histologic and immunophenotypic features centered around perivascular epithelioid cells.^1–5^ This tumor family includes angiomyolipoma, lymphangioleiomyomatosis, clear-cell ‘sugar' tumor, and PEComa not otherwise specified, and may arise in multiple anatomic sites including the uterus, kidney, gastrointestinal tract, retroperitoneum, lung, and liver.^4,6–8^

Primary hepatic PEComa is exceptionally rare. In our English-language literature review, earlier reports repeatedly noted that only approximately 30 hepatic cases had been described worldwide, which helps explain the persistent uncertainty surrounding diagnosis, prognostication, and treatment; more recent pooled reviews have identified larger cumulative numbers as additional case reports and retrospective analyses have emerged.^2,4,6,7,9^ Because of this rarity, most published evidence consists of case reports, small case series, retrospective analyses, and more recent systematic reviews, rather than formal guidelines.^3,6,7,9,10^

Noninvasive preoperative diagnosis is particularly difficult because hepatic PEComas have no pathognomonic clinical or radiological features. They often arise in noncirrhotic livers, frequently in women, and may show contrast enhancement patterns that closely resemble hepatocellular carcinoma (HCC) or other hypervascular hepatic lesions.^3,6,7,9,11^ Although many hepatic PEComas behave in an indolent fashion, malignant variants with recurrence, vascular invasion, metastasis, and death have been described, making accurate diagnosis and risk assessment clinically important.^2,3,6,10^

We report a large biopsy-proven primary hepatic PEComa in a young woman with prior colorectal cancer in remission, in whom the presentation raised strong suspicion for metastatic disease, highlighting the noninvasive diagnostic challenge posed by this rare tumor. The discordance between the extent of hepatic involvement and the patient's preserved clinical status was atypical for metastatic colorectal cancer, prompting reconsideration of the working diagnosis and prioritization of tissue confirmation before systemic therapy. This case highlights the importance of avoiding premature attribution of new liver lesions to recurrent malignancy and demonstrates the value of radiologic and pathologic correlation.

## CASE REPORT

A 28-year-old African woman with a history of colorectal cancer treated in her home country and in remission for 4 years presented to our institution with 3 months of nonradiating epigastric pain. She denied nausea, vomiting, postprandial exacerbation, altered bowel habits and weight loss. She had not been enrolled in a structured colorectal cancer surveillance program after her initial treatment.

Her reproductive history was notable for 2 pregnancies resulting in 2 children during the interval between colorectal cancer treatment and presentation to our facility. There was no documented pregnancy at presentation. Information regarding hormonal contraceptive use was not available in the medical record and was therefore not inferred retrospectively. She had no reported family history of malignancy. Mismatch repair, microsatellite instability, and germline testing had not been performed for the original colorectal cancer, and the original tumor tissue was not available at our institution for retrospective testing or pathological comparison.

On physical examination, she had tender, non-nodular hepatomegaly without jaundice, ascites, or splenomegaly. No other features of chronic liver disease were notable and the rest of the systemic examination was unremarkable.

Complete blood count showed hemoglobin 12.2 g/dL (normal range 12.0-15.0 g/dL), white blood cell count 4.28 ×10^9^/L (normal range 4.00-10.00 ×10^9^/L), and platelet count 240 ×10^9^/L (normal range 150.00-410.00 ×10^9^/L). Liver biochemistry demonstrated AST (67.40 U/L, normal range 0.00-34.00 U/L), ALT 325 U/L (normal range 0.00-49.00 U/L), GGT 324.00 U/L (normal range 0.00-38.00 U/L), ALP 325.00 U/L (normal range 46.00-116.00 U/L), total bilirubin 8.1 μmol/L (normal range µmol/L), and albumin 47.20 g/L (normal range 32.00-48.00 g/L). Renal function was preserved. Prebiopsy INR (1.06, normal range <2.00) was normal. Alpha-fetoprotein (1.75 ng/mL, normal range 0.00-7.00 ng/mL) and carcinoembryonic antigen (1.23 ng/mL, normal range 0-3.8 ng/mL) were within normal limits. Previous laboratory values from the patient's care in her home country were unavailable for comparison.

Ultrasound of the liver demonstrated 2 predominantly solid circumscribed heterogenous masses (Figure 1). One lesion in segment VII measured 7.3 × 7.8 cm. A second larger lesion extended across multiple inferior hepatic segments, involving segments IVb to VI, and measured 18 × 14 cm. The larger mass contained central anechoic areas and contributed to hepatomegaly. Triphasic abdominal CT demonstrated 2 large right hepatic lobe masses (Figure 2).

**Figure 1.**
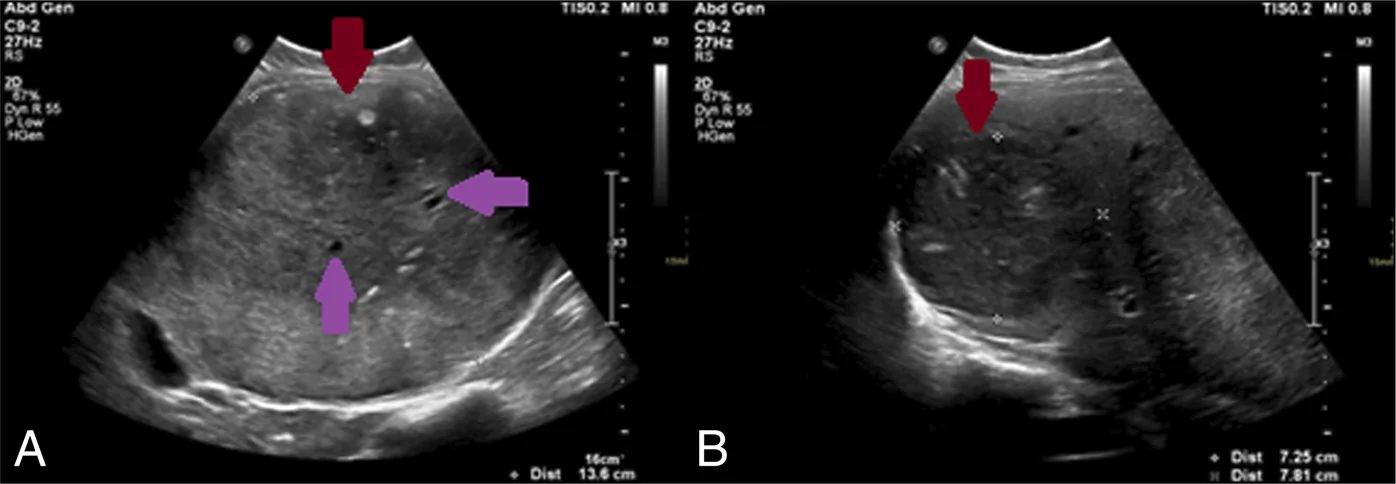
Ultrasound of the liver demonstrating 2 predominantly solid, circumscribed, heterogeneous hypoechoic masses (red arrows). (A) Shows a large lesion (18 × 14 cm) extending across segments IVb to VI, with central anechoic areas (purple arrows) and associated hepatomegaly. (B) Demonstrates a smaller similar lesion in segment VII, measuring 7.3 × 7.8 cm.

**Figure 2.**
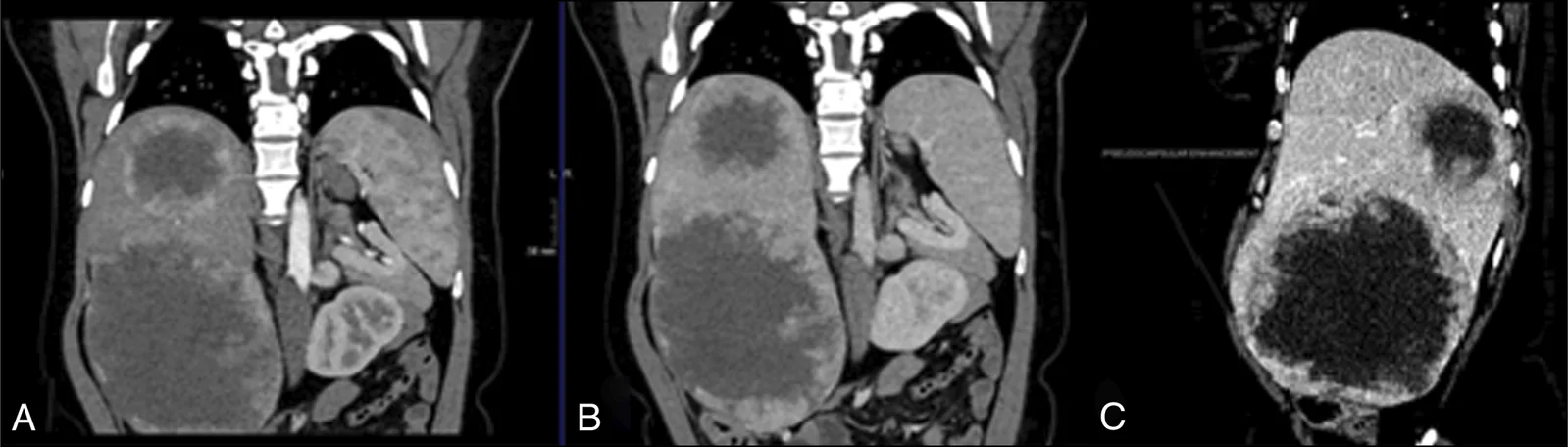
Triphasic abdominal computed tomography demonstrated 2 large right hepatic lobe masses. (A) On the arterial phase, both lesions showed avid peripheral heterogenous enhancement with central low-density components. (B) On the portal venous phase, there was progressive peripheral enhancement with peripheral pseudocapsular enhancement (better depicted in (C)).

Feeding arteries arising from the right hepatic artery were identified, together with early opacification of a draining vein into the right hepatic vein (Figure 3). Delayed imaging did not demonstrate significant washout (Figure 4).

**Figure 3.**
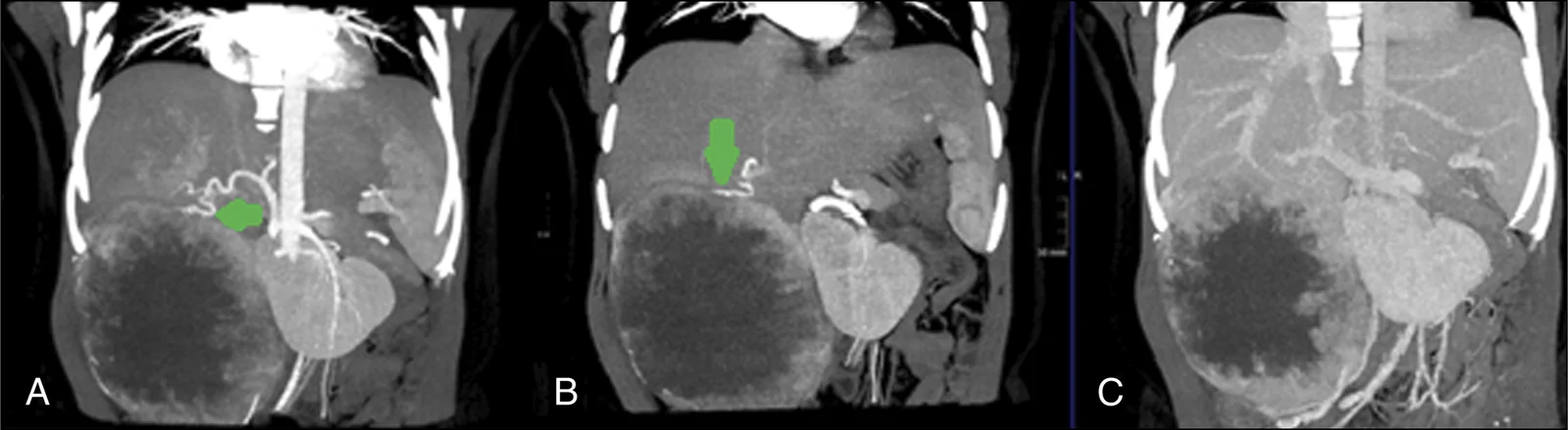
Feeding arteries from the right hepatic artery (green arrows) and early opacification of a draining vein into the right hepatic vein.

**Figure 4.**
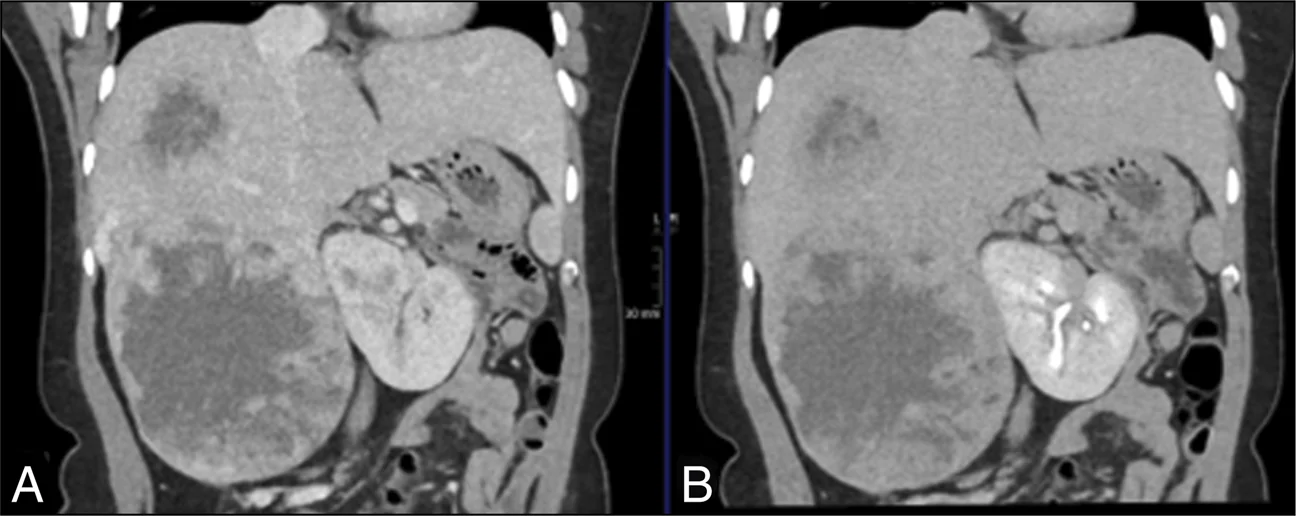
The delayed phase imaging did not show significant washout.

Given the patient's oncologic history and hypervascular nature of the lesions, the initial differential diagnosis included metastases, HCC, giant hemangioma, focal nodular hyperplasia, and other unusual primary hepatic tumors. The marked vascularity of the lesions raised concern regarding immediate percutaneous biopsy. Multiparametric liver MRI with hepatobiliary contrast was considered; however, hepatobiliary MRI contrast agents were not available locally at the time of evaluation. Following multidisciplinary discussion, hepatobiliary iminodiacetic acid scan (HIDA) SPECT-CT was performed as an available adjunctive functional hepatobiliary study to further characterize the hypervascular lesions, particularly to assess hepatobiliary tracer uptake in the context of focal nodular hyperplasia and other hypervascular hepatic mimics.

The HIDA scan demonstrated photopenic areas in the right lobe corresponding to the segment VII and segment IV-VI masses (Figure 5). Mild peripheral tracer uptake likely reflected surrounding hyperemia. No blood-pool activity or delayed tracer fill-in was demonstrated within the masses.

**Figure 5.**
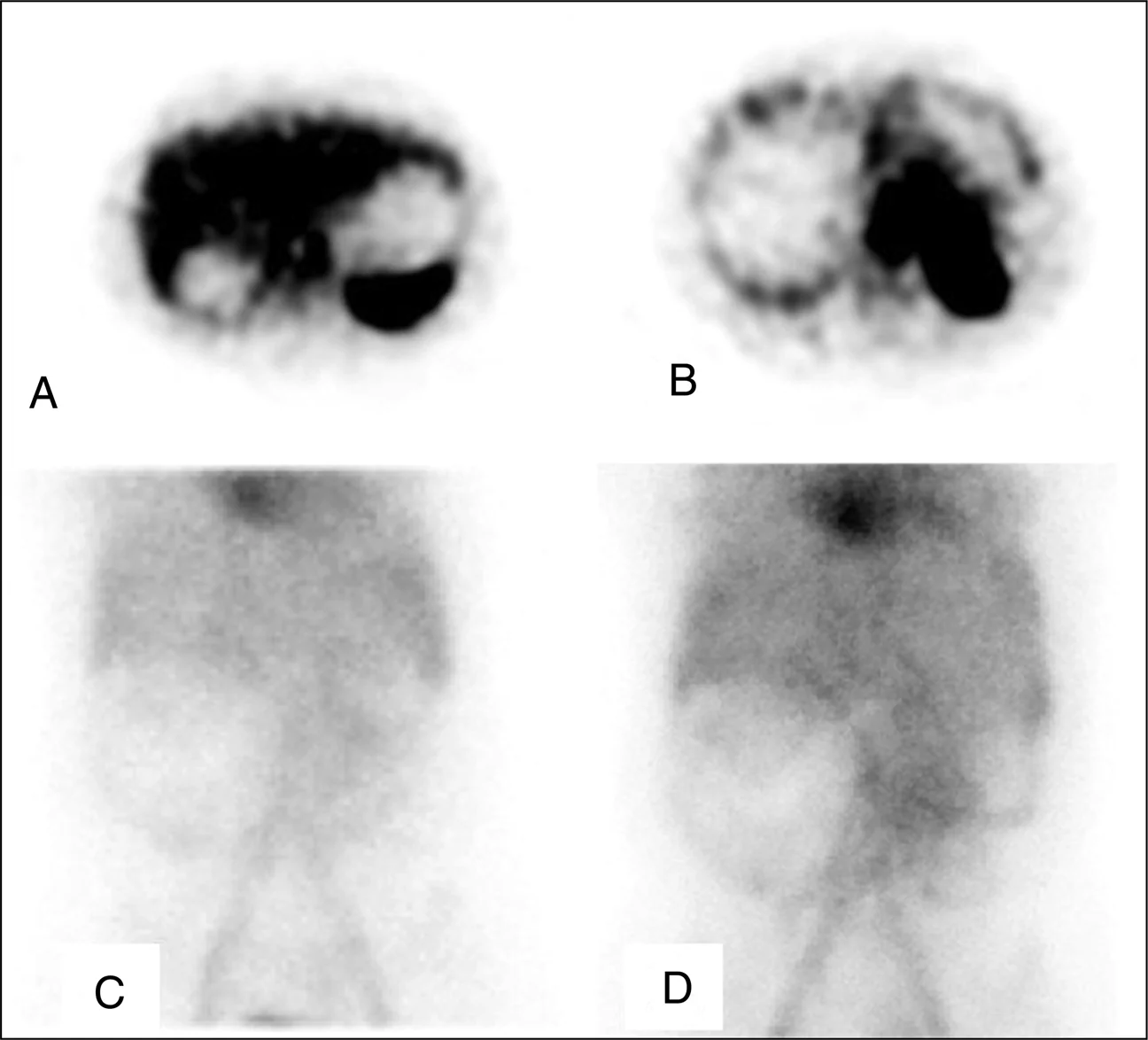
HIDA SPECT-CT demonstrating hepatomegaly with photopenic areas in the right hepatic lobe corresponding to masses in segments VII (A) and IV–VI (B). Mild peripheral tracer uptake is noted around the lesions, likely reflecting hyperemia. No blood pool activity (C) or delayed tracer fill-in (D) is seen within the masses.

In view of the inconclusive imaging findings, tissue diagnosis was performed. The procedure was not complicated by clinically significant bleeding, and there was no clinical evidence of hemorrhage or significant postprocedural hemoglobin decline. Fine-needle biopsy of the lesion showed hepatic tissue partly replaced by tumor arranged in cords and trabeculae. Tumor cells had moderately eosinophilic cytoplasm and nuclear pleomorphism. Preliminary pathology established a nonepithelial neoplasm, prompting extended immunohistochemical evaluation. The full immunohistochemical panel is summarized in Table 1. The final diagnosis was established 44 days after initial clinical presentation. Given the morphology and immunophenotype, the findings favored primary hepatic perivascular epithelioid cell tumor (PEComa; Figure 6). Additional immunohistochemical stains including S100, SOX10 and TFE3 were considered; however, these were not performed because of financial constraints, and available resources were prioritized toward the most diagnostically relevant stains after the lesion was already strongly leaning toward PEComa.

**Table 1.** Immunohistochemical profile of the hepatic lesion

Marker	Result
AE1/AE3	Negative
CK7	Negative
CK20	Negative
CDX2	Negative
HepPar1	Negative
Arginase	Negative
PAX8	Negative
LCA	Negative
Synaptophysin	Negative
CD56	Negative
SMA	Diffuse positive
HMB45	Focal positive
Ki-67	20%–30%
S100	Not performed
SOX10	Not performed
TFE3	Not performed

**Figure 6.**
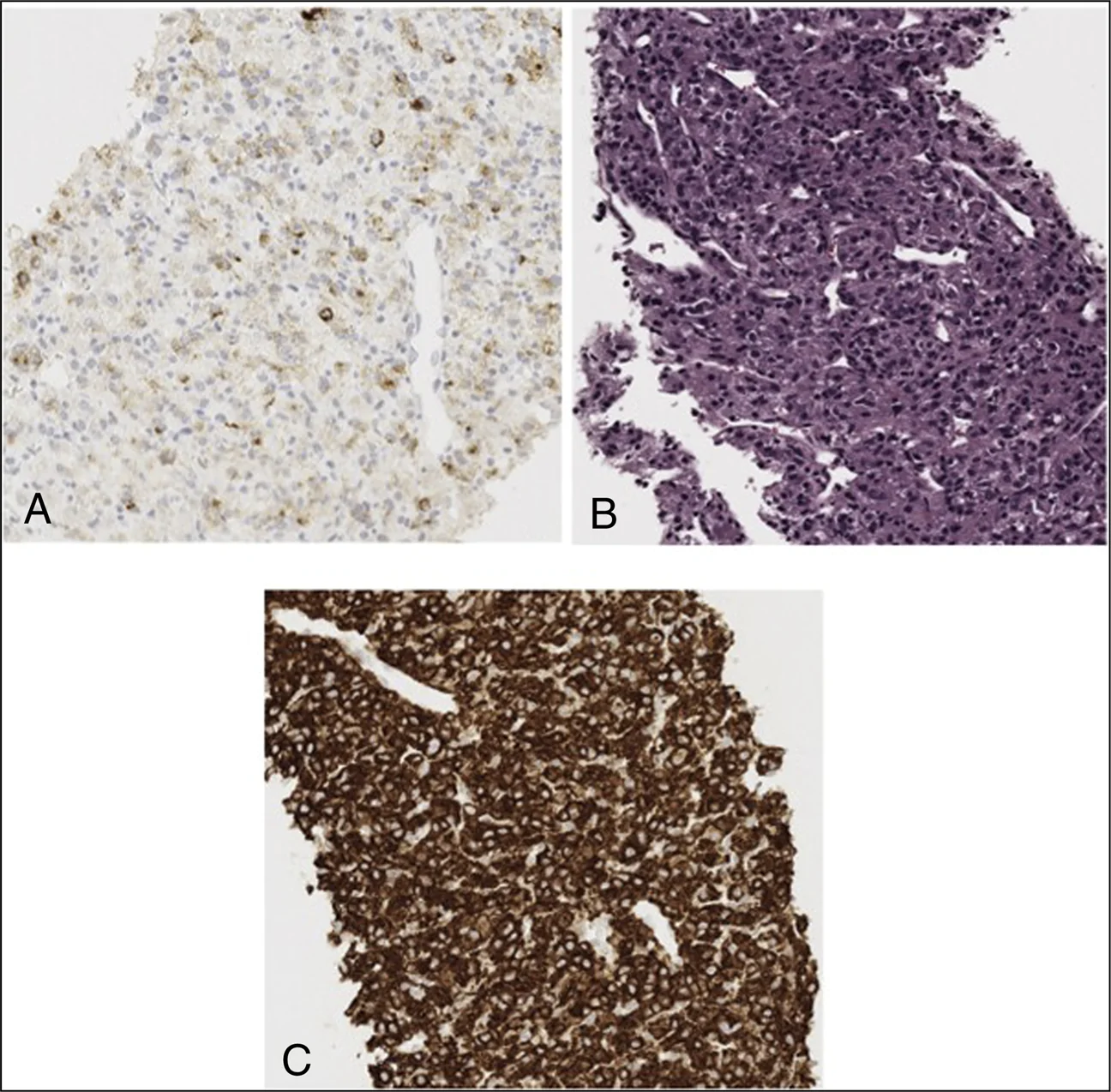
(A) HMB45 immunohistochemistry shows variable cytoplasmic staining in the tumor cells at ×200 magnification. (B) H&E staining shows cords and trabeculae of tumor cells with moderate pale to eosinophilic cytoplasm and mild to moderate nuclear pleomorphism at ×200 magnification. (C) Anti-smooth muscle antibody immunohistochemistry (smooth muscle marker) shows diffuse acytoplasmic positivity in the tumor cells at ×200 magnification.

The case was discussed in a multidisciplinary tumor board meeting. Locoregional therapy, including embolization of transarterial therapy, was considered during the multidisciplinary discussion because of the large size and vascularity of the lesions. However, after hepatobiliary surgical review confirmed that complete resection with negative margins was feasible, surgery remained the preferred definitive treatment. However, the patient requested additional time to organize financially before proceeding with surgery. In view of the tumor burden and concern for ongoing disease activity, everolimus 10 mg daily was commenced as interim therapy for disease control while she prepared for definitive surgical management. Everolimus was used as a temporary disease-control strategy while surgery was delayed, rather than a preferred alternative to resection. Unfortunately, the patient was lost to follow-up. Endoscopic surveillance was not current at presentation, and repeat colonoscopy and PET-CT could not be completed because of financial constraints.

## DISCUSSION

Primary hepatic PEComa remains diagnostically challenging because it combines extreme rarity with marked overlap in presentation and imaging appearance with more common liver tumors.^1–3,6^

The demographic and clinical profile of our patient mirrors several recurring features from the literature. Hepatic PEComa shows a strong female predominance, often arises in young or middle-aged adults, and commonly occurs in the absence of chronic liver disease.^3,4,6,7,10,11^ Our patient is also a woman and did not have an underlying liver disease.

The reproductive history is also relevant because a subset of PEComas has been reported to express estrogen and progesterone receptors, and case-level reports have described PEComa presentation or growth in pregnancy.^12^ Our patient had 2 pregnancies during the interval after treatment of colorectal cancer and before the current presentation. However, estrogen receptor and progesterone receptor testing were not performed on the hepatic tumor, contraceptive history was unavailable, and no causal relationship can be inferred.

Most patients are asymptomatic or present with nonspecific complaints such as abdominal pain, abdominal discomfort, nausea, upper abdominal fullness, or hepatomegaly once lesions become sufficiently large to exert mass effect.^4,6–8,11^ In this case, epigastric pain and tender hepatomegaly were likely attributable to the large tumor burden, especially the dominant lesion measuring 18 × 14 cm.

No laboratory test is specific for hepatic PEComa. Tumor markers such as AFP and CEA are mainly useful in excluding other lesions in the differential diagnosis rather than confirming PEComa itself.^6,7,11^ The normal AFP in our patient is particularly relevant because several studies emphasize that hepatic PEComa should be suspected when a lesion resembles HCC radiologically but arises in a noncirrhotic liver and lacks the expected clinical or serologic background of HCC.^2,3,6,11^

Radiologic diagnosis is difficult because lesion appearance reflects the variable proportions of adipose tissue, abnormal vessels, and smooth muscle cells within the tumor.^1,3,4,11,13^ On ultrasound, hepatic PEComas have been reported as hypoechoic, isoechoic, or hyperechoic masses, but they are often circumscribed and hypervascular on doppler imaging.^1,4,9,11^ Our patient's lesions were predominantly solid, circumscribed, and heterogeneous, which falls within this described sonographic range but remains nonspecific. The triphasic CT findings in this case are especially important. Hepatic PEComas commonly demonstrate marked arterial enhancement with variable persistence or washout in later phases, a pattern that can strongly mimic HCC.^1,3,4,6,11,13^ Both lesions in our patient showed avid peripheral arterial enhancement, progressive peripheral enhancement with a pseudocapsular appearance on the portal venous phase. However, 1 of the more helpful radiologic clues described in the literature is the presence of abnormal enlarged vessels, arteriovenous characteristics, and early draining veins, which may favor PEComa over HCC.^1,3,6,7,13,14^ That point directly applies here: feeding arteries from the right hepatic artery and early opacification of a draining vein into the right hepatic vein strongly suggested arteriovenous shunting and constitute 1 of the most distinctive imaging features of this case. The central low-density and anechoic areas within the larger lesion are also compatible with reported necrosis, hemorrhage, cystic degeneration, or heterogeneous internal architecture in larger hepatic PEComas.^3,4,11^

The range of radiologic differentials in this patient was broad. Her prior colorectal carcinoma made hypervascular metastases a legitimate concern. HCC was also a major consideration because of the arterial enhancement. Focal nodular hyperplasia and giant hemangiomas were relevant benign mimics in view of the patient's age and the hypervascular pattern. Nonetheless, the absence of cirrhosis, normal AFP and CEA, large abnormal tumoral vessels, early venous drainage, lack of classic centripetal fill-in, and lack of uptake on the HIDA scan collectively argued against these alternatives. Eventually, the histological immunophenotyping favored primary hepatic PEComa.^2–4,6,11,13,14^

The previous history of colorectal cancer also made recurrent or metastatic disease an important clinical concern. However, the normal CEA, atypical radiological appearance for colorectal metastases, absence of epithelial differentiation on biopsy, and negative AE1/AE3, CK20, and CDX2 staining argued strongly against metastatic colorectal adenocarcinoma. Repeat colonoscopy, PET-CT, and updated endoscopic surveillance would have further strengthened exclusion of recurrent colorectal cancer, but these could not be completed because of financial constraints.

As with most published hepatic PEComas, definitive diagnosis in this case depended on pathology and immunohistochemistry rather than imaging alone.^1,3,4,6,7,10^ Histologically, PEComas are characterized by epithelioid and/or spindle cells arranged around blood vessels, commonly in nests, lamellae, sheets, cords, or trabeculae, with clear to eosinophilic cytoplasm and variable atypia.^1,2,4,8^ The biopsy in our patient showed tumor cells arranged in cords and trabecular with moderate eosinophilic cytoplasm and nuclear pleomorphism, which fits well within the described morphologic spectrum.

Immunohistochemically, the hallmark of PEComa is expression of melanocytic markers such as HMB45 and Melan-A together with smooth muscle markers such as SMA, while markers supporting hepatocellular, cholangiocytic, or other epithelial differentiation are typically absent.^1,2,4,6,8,10,11^ Our patient's focal HMB45 positivity and diffuse SMA positivity strongly supported the diagnosis. This is particularly important because several papers in our review noted that not all tumors express every canonical marker diffusely and that focal staining does not exclude PEComa when the histologic context is appropriate.^1,2,13^ Additional markers reported in the literature include S100, vimentin, desmin, CD34, CD117, and TFE3, though their expression is variable and not required for diagnosis.^1,6,11,13^ The Ki-67 index of 20%-30% in this case is worrisome, since many benign hepatic PEComas in published series have lower proliferative indices.^2,3,6^ From a clinical perspective, an elevated proliferative index may be associated with more aggressive tumor behavior and increased risk of progression, although its prognostic value in PEComa remains incompletely defined.^2,3,6^ In this context, the Ki-67 finding contributed to the decision to pursue active management rather than observation, particularly given the substantial tumor burden.

The melanocytic immunophenotype also raises metastatic melanoma as a diagnostic consideration. S100 and SOX10 were not performed in this case, and this limits the completeness with which a melanocytic neoplasm could be excluded. However, the tumor morphology, focal HMB45 positivity, diffuse SMA positivity, and absence of epithelial, hepatocellular, colorectal, neuroendocrine, lymphoid, and renal/Müllerian markers supported PEComa in the appropriate clinicopathologic context.

Assessment of malignant potential remains 1 of the most difficult aspects of hepatic PEComa management. The most widely cited framework is the Folpe classification, which identifies tumor size greater than 5 cm, infiltrative growth, high nuclear grade, hypercellularity, necrosis, mitotic activity greater than 1 per 50 high-power fields, and vascular invasion as adverse features, with 2 or more favoring malignancy.^15^ Subsequent work, including the Bleeker analysis, emphasized tumor size and mitotic activity as especially important predictors of recurrence, and more recent hepatic-specific proposals have used size thresholds of at least 7 cm together with necrosis, infiltrative borders, vascular invasion, and PEComa-NOS phenotype as high-risk variables.^16^ In our patient, size alone is concerning, with lesions measuring 7.8 and 18 cm. Nuclear pleomorphism and the elevated Ki-67 index further heighten concern. At the same time, limited biopsy material cannot fully assess infiltrative growth, overall mitotic burden, vascular invasion, or coagulative necrosis to the same degree as a resection specimen, and some reports have specifically cautioned that malignant potential may be underestimated on biopsy alone.^6,14^ Although formal risk stratification using these frameworks was not applied in this case, key high-risk features, including large tumor size and elevated proliferative index were considered clinically significant. In the absence of a resection specimen, a pragmatic, oncology-driven approach to risk assessment was adopted, supporting active management rather than observation.

The molecular background of PEComa also has therapeutic implications. Several studies have linked PEComa pathogenesis to dysregulation of the mTOR pathway, often through TSC1 or TSC2 abnormalities, which provides a rationale for targeted therapy even in sporadic cases without obvious tuberous sclerosis complex.^1,2,6,7,11,13^ TFE3-rearranged PEComas are increasingly recognized as a distinct subgroup, often occurring in younger patients and showing strong TFE3 and HMB45 expression with reduced smooth muscle marker expression, although TFE3 status was not available in our patient.^1,2,6^ Our literature review also noted that some reports have described associations with TSC and, more recently, rare cases linked to other genetic syndromes such as Li-Fraumeni syndrome, underscoring the evolving molecular landscape of this tumor group.^6^ In our case, genetic testing for TSC was not undertaken due to financial constraints. In addition, TFE3 immunohistochemistry and molecular testing were not performed, limiting molecular subclassification.

Treatment of hepatic PEComa remains controversial because of the rarity of the disease, but surgical resection with negative margins is still regarded as the treatment of choice for localized lesions and is associated with the best documented outcomes.^1–4,6,7,10,11^ That principle applied directly in this case, where hepatobiliary surgical review confirmed that complete resection with negative margins was feasible and safe. Alternative or adjunctive modalities described in the literature include surveillance for selected small benign-appearing lesions, radiofrequency ablation, embolization, TACE, stereotactic body radiotherapy, and systemic mTOR inhibition in advanced, unresectable, metastatic, or neoadjuvant settings.^1,3,4,6,7^ Locoregional therapy was considered during multidisciplinary review; however, after the lesions were deemed resectable, surgery remained the preferred definitive treatment. In our patient, surgery was deferred temporarily for financial reasons rather than technicality of resectability. Given the lesion size, multifocality, and proliferative index, observation alone was not ideal during that interval. Everolimus was therefore initiated as interim disease control. This strategy is biologically plausible and literature-supported, as mTOR inhibitors such as sirolimus, everolimus, and nab-sirolimus have demonstrated activity in malignancy or unresectable PEComa and, in some reports, have enabled subsequent surgery after tumor shrinkage.^3,4,6,7^ From a clinical perspective recognition of mTOR pathway activation provided a biologic rationale for the use of targeted therapy in this case. Although molecular confirmation was not available, treatment decisions were guided by established disease biology and available evidence, supporting the use of mTOR inhibition as a pragmatic therapeutic approach. In this setting, everolimus functioned as a bridging rather than definitive therapy while curative resection remained the intended endpoint.

Overall, this case reinforces several practical messages including diagnostic and management challenges of primary hepatic PEComa. Although rare, hepatic PEComa should be considered in the differential diagnosis of noncharacteristic hypervascular liver lesions in young women with normal AFP, noncirrhotic liver, abnormal enlarged tumoral vessels and early venous drainage. The definitive diagnosis still depends on histopathology and immunohistochemistry.

This case highlights a rare primary hepatic perivascular epithelioid cell tumor presenting as large hypervascular liver masses in a young woman with prior colon cancer, a noncirrhotic liver and normal tumor markers. The diagnosis was challenging due to significant overlap with more common hepatic lesions, particularly in the context of a prior malignancy. Ultimately, accurate diagnosis was achieved through radiologic-pathologic correlation, with immunohistochemistry demonstrating melanocytic and smooth muscle differentiation.

Multidisciplinary team involvement was central to management planning, confirming that complete surgical resection with negative margins was feasible. Interim treatment with everolimus was initiated as a bridging strategy while the patient prepared for surgery, reflecting the emerging role of mTOR-targeted therapy in selected cases.

Although hepatic PEComa often demonstrates favorable outcomes following resection, its biological behavior remains unpredictable, particularly in the presence of large tumor size, nuclear pleomorphism, and elevated proliferative index. Unfortunately, the patient was subsequently lost to follow-up, and therefore clinical outcome could not be determined.

## LIMITATIONS

A key limitation of this report is the loss to follow-up after initiation of interim therapy, which precluded assessment of treatment response, surgical outcome, and long-term prognosis. In addition, lack of complete surgical pathology limited full evaluation of malignant risk features such as vascular invasion and mitotic activity. Molecular profiling was not performed, limiting correlation with underlying genetic alterations and potential therapeutic targets. Further limitations include the absence of multiparametric liver MRI, PET-CT, repeat colonoscopy, updated endoscopic colorectal cancer surveillance, S100, SOX10, TFE3, estrogen receptor/progesterone receptor testing, MMR/MSI testing, and germline testing. Several of these investigations could not be completed because of financial constraints; additional immunohistochemistry was also limited by cost, with testing prioritized toward markers most likely to resolve the immediate diagnostic question. The MRI could not be performed as the hepatobiliary MRI contrast agents were not available locally at the time of the patient's evaluation.

## DISCLOSURES

Author contributions: G. Odong, P. Panwar, and L. Otom were involved in the drafting and write-up of the case report. G. Odong provided the radiological images and interpretation. S. Idet, S. Kibe, and I. Wakahora provided the radiological images and contributed to their interpretation. P. Okiro provided the pathology images and related diagnostic input. A. Alemanji and A. Kidee were involved in the primary clinical care of the patient. S. Gitau and S. Abdulkarim supervised the manuscript preparation and critically reviewed the final manuscript. All authors reviewed and approved the final version of the manuscript. S. Gitau is the article guarantor.

Financial disclosure: None to report.

Informed consent was obtained for this case report.

## ABBREVIATIONS:

AE1/AE3: Pancytokeratin antibody cocktail
AFP: Alpha-fetoprotein
ALP: Alkaline phosphatase
ALT: Alanine aminotransferase
ASMA: Alpha-smooth muscle actin
AST: Aspartate aminotransferase
CD117: Cluster of differentiation 117
CD34: Cluster of differentiation 34
CD56: Cluster of differentiation 56
CDX2: Caudal type homeobox 2
CEA: Carcinoembryonic antigen
CK20: Cytokeratin 20
CK7: Cytokeratin 7
CT: Computed tomography
GGT: Gamma-glutamyl transferase
H&E: hematoxylin and eosin
HCC: hepatocellular carcinoma
HepPar1: Hepatocyte paraffin 1
HIDA: Hepatobiliary iminodiacetic acid
HMB45: Human melanoma black 45
INR: International normalized ratio
Ki-67: Ki-67 cell proliferation marker
LCA: Leukocyte common antigen
MMR: Mismatch repair
MRI: Magnetic resonance imaging
MSI: Microsatellite instability
mTOR: Mechanistic target of rapamycin
PAX8: Paired box 8
PEComa: Perivascular epithelioid cell tumor
PEComa-NOS: Perivascular epithelioid cell tumor not otherwise specified
PET-CT: Positron emission tomography–computed tomography
S100: S100 calcium-binding protein
SMA: Smooth muscle actin
SOX10: SRY-box transcription factor 10
SPECT-CT: Single-photon emission computed tomography–computed tomography
TACE: Transarterial chemoembolization
TFE3: Transcription factor binding to IGHM enhancer 3
TSC: Tuberous sclerosis complex
TSC1: TSC complex subunit 1
encodes hamartin: TSC2
TSC complex subunit 2: encodes tuberin
